# Proanthocyanidin Structure-Activity Relationship Analysis by Path Analysis Model

**DOI:** 10.3390/ijms24076379

**Published:** 2023-03-28

**Authors:** Zhaoxuan Li, Jingling Liu, Jie You, Xin Li, Zongsuo Liang, Junli Du

**Affiliations:** 1 College of Sciences, Northwest A&F University, Yangling 712100, China; 2 College of Life Sciences, Northwest A&F University, Yangling 712100, China; 3 College of Life Sciences and Medicine, Zhejiang Sci-Tech University, Hangzhou 310018, China

**Keywords:** proanthocyanidins, structure-activity relation, path analysis, *Rhodiola crenulata*

## Abstract

To fully explore the influence mechanism of interactions between different monomer units of proanthocyanidins (PAs) on biological activity, a path analysis model of the PA structure-activity relationship was proposed. This model subdivides the total correlation between each monomer unit and activity into direct and indirect effects by taking into account not only each monomer unit but also the correlation with its related monomer units. In addition, this method can determine the action mode of each monomer unit affecting the activity by comparing the direct and total indirect effects. Finally, the advantage of this model is demonstrated through an influence mechanism analysis of *Rhodiola crenulata* PA monomer units on antioxidant and anti-diabetes activities.

## 1. Introduction

Proanthocyanidins (PAs), also referred to as condensed tannins, are mainly composed of flavan-3-ol monomer units, i.e., epicatechin, catechin, and/or epicatechin-3-O-gallate, etc., through C4-C8 or C4-C6 bonds, and represent the most abundant class of natural phenolic compounds [1,2,3] (Appeldoorn et al., 2009; Hümmer & Schreier, 2008; Liu et al., 2018). PAs are occurred naturally in roots, leaves, flowers, fruits, and seeds of a wide variety of different edible plants, with varying compositions and percentages of monomeric catechins, epicatechin, oligomers, and polymers [1,3]. The structures of PAs are made complicated by the multiple permutations of monomeric flavan-3-ol units and various types of linkages among them, leading to large numbers of theoretically existing isomers [4]. Importantly, a number of bioactivities and pharmacological effects have been reported for PAs, for example, antioxidant activity [5], anti-inflammatory [6], antibacterial [7], anticancer [8], antiviral [3,9], and anti-aging effects [10]. Because of these beneficial health effects and the prevention of various diseases, PAs have attracted more and more research interest.

In recent years, there are an increasing number of studies reported in the literature that explored the PA structure-activity relationship. For instance, Cedó et al. pointed out that there is a relationship between anti-diabetic activity and the structure of PAs in grape seeds [11]. Ge et al. found that the antioxidant activities of type-A and type-B procyanidin dimers changed oppositely after incubation with rat intestinal microbiota for 6 h in vitro [12]. Joshi et al. found that polymeric procyanidins showed higher antiviral effects than monomeric catechins [13]. Zhou et al. determined that highly polymerized procyanidins usually possessed lower antioxidation and greater anti-digestion properties than oligomers [14]. In order to find the structural features responsible for the activities, Alejo-Armijo A. et.al designed and synthesized six A-type procyanidins and evaluate their antimicrobial and antibiofilm properties against 12 resistant bacteria [15]. Zhang et al. thoroughly investigate the structure, degree of polymerization, and starch hydrolase inhibition activities of bird cherry PAs, indicating that bird cherry oligomeric PAs are a promising starch hydrolase inhibitor for the application of potential functional food components [16]. Xu et al. studied the effects of EGCG, EGC, and ECG on the chemical and cell-based antioxidant activity, sensory properties, and cytotoxicity of a catechin-free model beverage using response surface methodology [17]. 

In short, previous studies have shown that the biological activities of PAs are highly dependent on their structure [18]. Currently, some researchers have proposed methods to quantitatively study the structure-activity relationship, for example, QSAR, the isobole method, the response surface method, etc. QSAR is an effective method to measure the relationship between the structure and biological effects of a single compound by analyzing the influence of structural changes on biological effects. This allows candidate molecules with more balanced properties to be designed. The model and its improved model focus more on predicting activity by structure [19,20,21,22,23]. However, when more than two compounds are present at the same time, the activities may not be additive due to the interactions caused by hydrogen bonds or steric effects between multiple components. This fact indicates that the interaction (i.e., correlation) between different compounds produces a combined effect on activity. The mechanisms underlying interactions should be known for the standardization and optimization of mixtures of different compounds and also for the formulation of single extracts or multi-extract preparations [24]. The isobole method is widely utilized to describe and prove combined effects for multiple component mixtures like herbal mixtures or plant extracts. However, this method is independent of the mechanism of action [25]. The response surface method is widely used to study the isolated and binary/ternary effects of chemical compounds to obtain the optimal combination of chemical compounds, which benefits the development of mixtures containing bioactive compounds to be further incorporated into medicine with enhanced effects [17,26]. Unfortunately, due to the interaction between different structures in the phenolic compound mixture, the influence mechanism of the structure on activity has not been fully comprehended, although researchers attempt to find these interactions by experiment [17]. In fact, currently, the influence of interaction between structures on activity is hidden behind the structural characterization data and activity data. In order to more accurately and comprehensively explore the complex influence mechanism of PA structure on activity and give consideration to interactions between PAs, there is an urgent need to seek a statistical method for the quantitative study of the structure-activity relation of PAs based on the structure and bioactivity data of PAs collected by existing technical means. The path analysis model proposed by Wright (1921) can be used to analyze the detailed influence mechanism of independent variables with interaction on dependent variables and has been successfully applied to gene function analysis based on the KEGG pathway database [27].

*Rhodiola crenulata* is a traditional herbal medicine that originated in Eastern Europe and Asia. The root extracts of *Rhodiola crenulata* contain polyphenols such as flavonoids and proanthocyanidines [28]. Previous research studies have shown that *Rhodiola crenulata* can not only prevent and treat acute mountain sickness but also possesses a variety of bioactivities, including anti-diabetes, anti-fatigue, antidepressant, antioxidant, anti-inflammatory, and anticancer activities [29,30]. However, up to now, there has been no research on the relationship between monomer units and the activity of *Rhodiola crenulata*.

In this paper, we aim to establish a path analysis model for the structure-activity of PAs to explore the influence mechanism of PA monomer units on activity. In this model, the importance of each monomer unit to the activity is determined by the order of the total correlation coefficient. At the same time, the influence mechanism of each monomer unit on the activity is revealed by subdividing the total correlation coefficient into its own direct effect and indirect effect with other monomer units. Finally, the utility of the model is demonstrated using the data of monomer units of *Rhodiola crenulata* PAs and the data of antioxidant and anti-diabetes activities.

## 2. Results

### 2.1. The Results of Monomer Units and Antioxidant Activities Analysis

Firstly, the F test showed that the standardized empirical linear regression equation between the monomer units of *Rhodiola crenulata* PAs and antioxidant activity was extremely significant (p≤0.01). And the value of the determination coefficient $R^{2}$ was 0.9934. This phenomenon indicated that the monomer units can determine 99.34% of the variation in antioxidant activity. 

**Figure 1:**
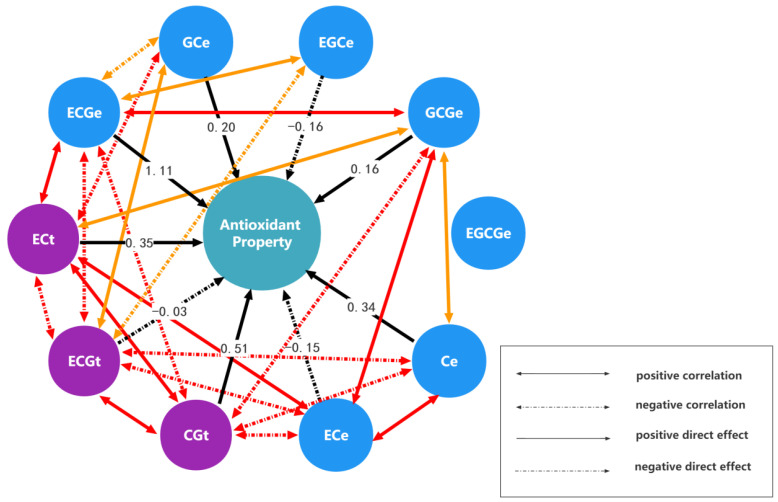
The completely closed path chart of *Rhodiola crenulata* proanthocyanidin monomer units and antioxidant activity. (Note: The correlation coefficient of the red correlation path in the figure is greater than or equal to 0.85, while the correlation coefficient of the orange correlation path ranges from 0.75 to 0.85).

Next, a path analysis of monomer units and antioxidant activity was conducted according to path analysis theory (Section 4.1). The detailed subdivision results of the path analysis are listed in Table 1. In order to display the influence mechanism of different monomer units on the antioxidant activity more intuitively, a path diagram of monomer units and antioxidant activity was drawn (Figure 1). It should be noted that in order to make the path diagram clear, only the correlation paths with correlation coefficients greater than or equal to 0.7 are drawn in Figure 1. 

**Figure 2:**
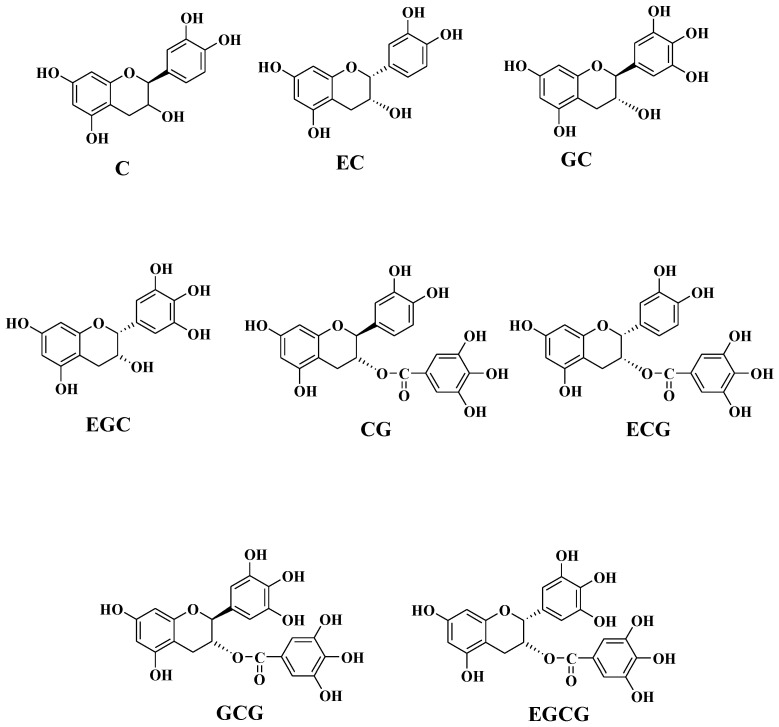
Chemical structures of investigated polyphenolic compounds C, EC, GC, EGC, CG, ECG, GCG, and EGCG.

As shown in Table 1, the correlation order of monomer units and antioxidant activity is ECGt > ECGe > CGt > ECt > Ce > GCGe > ECe > GCe > EGCe > EGCGe in aqueous solution. As shown in Figure 1, the monomer units that seem to be closely related to the antioxidant activity are also more related to other monomer units. Moreover, it is obvious that monomer units ECGt and ECGe have the strongest correlation with antioxidant activity. This result is consistent with the fact that the correlation between ECG from green tea extracts and antioxidant activity is the strongest in aqueous solution [31]. A previous study also suggested that the order of the antioxidant activity of four green tea epicatechins was ECG > EC > EGC in oil-in-water emulsions [32]. This result is still consistent with the correlation order we obtained. However, it is strange that ECGt has a negative correlation (−0.9822) with antioxidant activity, but ECGe has a positive correlation (0.9716) with antioxidant activity. This phenomenon shows that the same monomer unit has different effects on the antioxidant activity at different positions. From the subdivision results, the direct effect of ECGe (1.1117) is much greater than the indirect effect from other monomer units. This result showed that the positive correlation between ECGe and antioxidant activity is mainly caused by the direct effect of ECGe. Conversely, the direct effect of ECGt (−0.0274) is very small. This result showed that the strong negative correlation between ECGt and antioxidant activity is mainly caused by stronger indirect regulation of ECGt from the other monomer units. In particular, the indirect regulation of ECGe on ECGt is up to −1.1053. In fact, it can be seen from the penultimate row of Table 1 that the indirect regulation of ECGe on monomer units other than itself is relatively large. Therefore, whether directly or indirectly regulated, ECGe is very important for antioxidant activity. For ECGt, although the total correlation coefficient with antioxidant activity is greater than that between ECGe and antioxidant activity, its direct effect on antioxidant activity and indirect regulation to other monomer units are relatively small. Hence, it seems that ECGe is more important than ECGt from the subdivision results. CGt is also closely related to antioxidant activity, perhaps because it has a similar structure to ECG, both containing one pyrogallol and one catechol [33]. The direct effect of CGt on antioxidant activity is moderate, but the total effect is still mainly caused by indirect regulation. The correlation between the monomer unit ECt and antioxidant activity was slightly weak. From the subdivision of the total effect, there is little difference between the direct effect (0.3521) and the indirect effect (0.5861). The results demonstrated that the direct effect and indirect effect together lead to the influence of ECt on antioxidant activity. The correlation between Ce and antioxidant activity and its influence on antioxidant activity is similar to ECt, perhaps because they have a similar structure including one catechol (Figure 2). It should be noted that the correlation between ECe and antioxidant activity has decreased and the influence mode has changed. The direct effect (−0.1534) is far less than the indirect effect (1.01). The monomer units GCe and EGCe have little difference in their effects on antioxidant activity and their influence modes are mainly indirect regulation. The direct effect of monomer unit GCGe (0.1596) is small. However, the strong indirect effect leads to a greater correlation between it and antioxidant activity. The correlation between monomer unit EGCGe and antioxidant activity is the smallest. The direct effect of EGCGe is almost zero (−0.0031), and the total correlation is almost completely caused by the indirect regulation of other monomer units. Such results contradict reports in the literature that the effectiveness of EGCG is second only to that of ECG in terms of antioxidant activity [31]. This result may be due to the small content of EGCG in that the previous research pointed out that the antioxidation of phenolic compounds depends not only on their structures but also on their concentrations [24]. 

### 2.2. The Results of Monomer Units and Anti-Diabetes Bioactivities Analysis 

Firstly, the standardized empirical linear regression equation between the monomer units of *Rhodiola crenulata* PAs and anti-diabetes bioactivity was also extremely significant under the F test (p≤0.01). The value of the determination coefficient $R^{2}$ was 0.9949. This phenomenon indicated that the monomer units can determine 99.49% of the variation in anti-diabetes bioactivity. 

**Figure 3:**
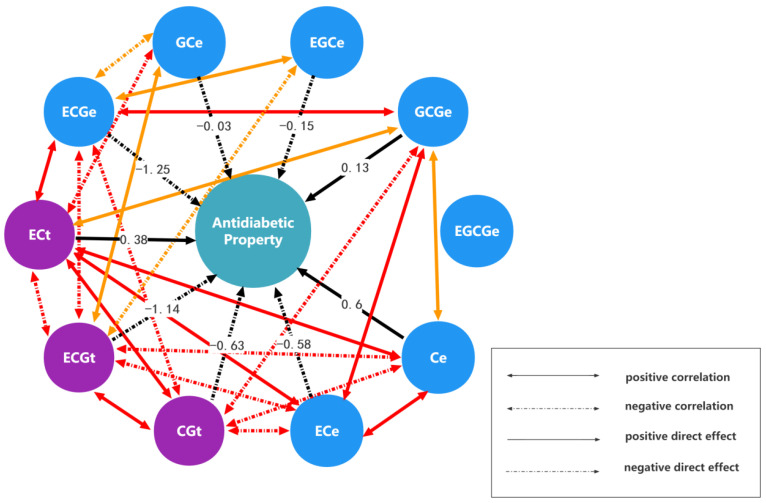
The completely closed path chart of *Rhodiola crenulata* proanthocyanidin monomer units and anti-diabetic activity. (Note: The correlation coefficient of the red correlation path in the figure is greater than or equal to 0.85, while the correlation coefficient of the orange correlation path ranges from 0.75 to 0.85).

Next, a path analysis of monomer units and anti-diabetes bioactivity was conducted according to path analysis theory (Section 4.1). Table 2 lists the detailed subdivision results of the path analysis. Figure 3 displays the influence mechanism of different monomer units on the anti-diabetes activity more intuitively, where only the correlation paths with correlation coefficients greater than or equal to 0.7 are drawn. 

It can be seen from Table 2 that the correlation order of monomer units and anti-diabetes activity is Ce > ECt > ECGt > CGt > ECGe > ECe > GCe > GCGe > EGCe > EGCGe in aqueous solution. As shown in Figure 3, the correlations of monomer units closely related to anti-diabetes activity with the other monomer units are also stronger. 

In detail, Ce and ECt have the strongest positive correlation with anti-diabetes activity. This result is consistent with the conclusion that C and EC in green tea can enhance anti-diabetes activity by inducing a significant reduction in cellular glucose uptake in Na+ -dependent conditions [34]. According to the total correlation subdivision, the direct effect of Ce (0.6063) is greater than the total indirect effect (0.3617), which indicates that Ce mainly directly regulates anti-diabetes activity. Differently, the total indirect effect of ECt (0.5803) is greater than the direct effect (0.3837), which indicates that ECt mainly indirectly regulates anti-diabetes activity through the other monomer units. CGt and ECGt showed a stronger negative correlation with anti-diabetes activity (−0.9004 and −0.9109), which may be because they are isomers (Figure 2). From the subdivision, the direct effect of CGt and ECGt (−0.627 and −1.1364, respectively) is much greater than the indirect effect (−0.2734 and 0.2255, respectively). The results showed that CGt and ECGt mainly directly regulate anti-diabetes activity. In addition, it is gratifying that Ce has a large negative indirect effect on CGt and ECGt, which is consistent with the conclusion that green tea catechins (C) have a significant inhibitory effect on epicatechin gallate (ECG) [35,36]. Strangely, unlike ECGt, ECGe is positively correlated with anti-diabetes activity, and its direct effect on anti-diabetes activity (−1.2508) is far less than its indirect effect (2.1308), which demonstrated that the effect of the same monomer unit on anti-diabetes activity is different in different positions. The monomer units ECt and ECe are both positively correlated with anti-diabetes activity and are mainly indirectly regulated, but it is obvious that the regulation effect of ECt is greater. The monomer units GCe, EGCe, and GCGe have a medium influence on anti-diabetes activity. Their direct effect on anti-diabetes activity is relatively small, indicating that these monomer units mainly indirectly regulate anti-diabetes activity through other monomer units. It should be pointed out that the indirect effects of ECGt and ECGe are all greater. The total correlation between EGCGe and anti-diabetes activity is minimal and the total indirect effect (−0.2898) is almost equal to the total effect (−0.2828), which showed that the effect of EGCGe on anti-diabetes activity is the smallest and also mainly indirect. However, the indirect effect of EGCGe on anti-diabetes activity through ECGe (0.7188) is large and positive. The result is consistent with the result reported in the literature that ECG and EGCG were shown to inhibit SGLT1 (Sodium-Glucose Co-Transporter)-mediated glucose uptake [34,35]. 

### 2.3. The Comparison of Monomer Units on Antioxidant and Anti-Diabetes Activities Analysis Result

In order to explore the differences in the effects of different monomer units on antioxidant and anti-diabetes activities, we made a comparison (Table 3). It can be seen from the results in Table 3 that the monomer units affecting the two activities can be divided into three categories according to the importance of the effect on the activities. The first category includes ECGt, ECGe, CGt, ECt, and Ce, in which the total effect of ECGe on anti-diabetes activity is 0.88, and the other total effects are above 0.9. The second category includes GCGe, ECe, and GCe. The total effect of these monomers on the two activities ranges from 0.77 to 0.88. The remaining monomers, EGCe and EGCGe, belong to the third category. Their total effect on both activities is less than 0.7, and even the total effect of EGCGe and anti-diabetes activity is only −0.2828. It is obvious that the influence modes of the second- and third-category monomer units on the activity are mainly indirect regulation.

The impact of monomer units in the first category on antioxidant and anti-diabetes activities is quite different. For ECGt and ECGe, their correlation with antioxidant activity is greater than their correlation with anti-diabetes activity. Moreover, ECGt was negatively correlated with antioxidant and anti-diabetes activities. Conversely, ECGe was positively correlated with both activities. From the perspective of influence mode, the influence of ECGt on antioxidant activity is mainly indirect regulation, but the influence on anti-diabetes activity is mainly direct regulation. In contrast, the effect of ECGe on antioxidant activity is mainly direct, while the effect on anti-diabetes activity is mainly indirect. These results show that the same monomer unit in different positions (extension unit or terminal unit) has different effects on the activity. The effect of CGt on anti-diabetes and antioxidant activity is the same as that of ECGt in magnitude and manner, which may be because the enantiomers CGt and ECGt are located in terminal units and do not have structural differences (i.e., in an achiral environment) (Figure 2). As shown in Table 3, for ECt and Ce, their correlation with anti-diabetes activity is greater than their correlation with antioxidant activity, and they are positively correlated with both activities. In addition, ECt mainly affects antioxidant and anti-diabetes activities indirectly, but the impact of Ce on antioxidant activity is mainly indirect, and the influence on anti-diabetes activity is mainly direct.

ECt and ECe are in different positions, but their effects (positive correlation regulation) and influence modes (mainly indirect) on activity are the same. Conversely, ECGt and ECGe are also in different positions, but they have different effects on the two activities in different directions and influence modes. This result seems to indicate that gallate plays a role in the process of affecting the activity (Figure 2), which is consistent with the results of a study of green tea polyphenols [36].

## 3. Discussion

In this study, a path analysis model is firstly proposed to analyze the structure-bioactivity relation of PAs. Unlike other common approaches to quantitative structure-activity relationship analysis, it focuses more on quantifying the effect of interactions between various monomer units on activity. In addition, the influence mechanisms of interactions between different monomer units on activity can be displayed by subdividing the total correlation (total effect) between each monomer unit and activity into direct and indirect effects. This subdivision is realized by the canonical equations in regression analysis. One highlight of the path analysis model is that it quantifies the effect of interaction caused by the correlation between different monomer units on the activity, which is not only conducive to the intuitive comparison and understanding of the influence mechanism but also provides inspiration and reference for the development and utilization of proanthocyanidin resources. The path analysis results of the structure-activity relation of *Rhodiola crenulata* PAs demonstrate that this method not only can produce more biologically meaningful results proved by previous literature but can also clearly and intuitively display the complex regulatory mechanism of different monomer units on antioxidant and anti-diabetes activities through path analysis tables (Table 1 and Table 2) and path charts (Figure 1 and Figure 3). Hence, the path analysis model of the structure-activity relationship of PAs is a meaningful and valuable addition to structure-activity relationship of PAs analysis methodology.

The comparison analysis of *Rhodiola crenulata* PA monomer units on antioxidant and anti-diabetes bioactivities showed that the main monomer units affecting antioxidant and anti-diabetes activities are ECGt, ECGe, CGt, ECt, and Ce. Moreover, these monomer units have more influence on antioxidant activity than on anti-diabetes activity. This may be because anti-diabetes activity itself is related to antioxidant activity. However, the modes of influencing the two activities are quite different. For antioxidant activity, other monomer units mainly affect the activity by indirect correlation regulation between monomers other than ECGe. However, ECGt, CGt, and Ce mainly affect the anti-diabetes activity by direct effect. In addition, the position (extension unit or terminal unit) of the monomer unit will affect the direction and mode of the monomer unit affecting the activity, such as in the case of ECGt and ECGe. 

Currently, the proposed model mainly focuses on the study of the structure-activity relationship between PAs monomer units and activity. Considering more information, such as linkage types, degree of polymerization, and the correlation between different activities, is still a challenge for exploring the structure-activity relationship of Pas. In order to better understand the structure-activity relation of Pas, addressing these issues, coupled with technological advances, will likely improve confidence in the results.

## 4. Methods and Materials

### 4.1. The Path Analysis Model for Structure-Activity Relation of PAs

The path analysis model of the structure-activity relationship of PAs is essentially a standardized multiple linear regression analysis. The novelty of the model is to deeply explore the influence mechanism of PA monomer units on bioactivity by subdividing the total correlation between PA monomer units and bioactivity. 

**Figure 4:**
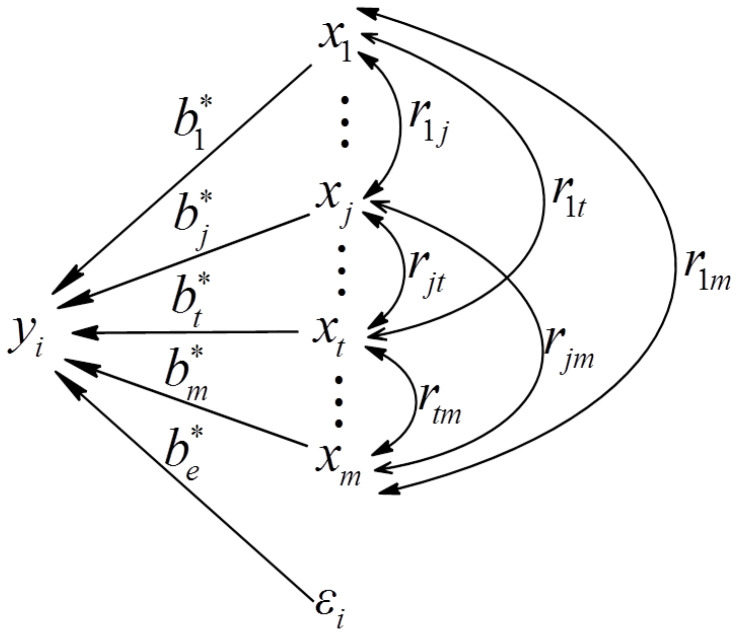
The completely closed path chart of PAs structure-activity with independent error.

To introduce the path analysis model of the structure-activity relationship of PAs, we define the following notations. Let $x={\left(x_{1},x_{2},x_{3},\ldots ,x_{m}\right)}^{T}$ be the set of PA monomer units and $y_{i}$ be the value of PA activity. The vector x is assumed to follow a normal distribution, $x∼N(0,R_{xx})$, where $R_{xx}$ is the correlation matrix of x. Let ${y^{\prime }}_{i}^{}$ and ${x^{\prime }}_{j}^{}\left(j=1,2,\cdots ,m\right)$ denote the standardized $y_{i}$ and $x_{j}\left(j=1,2,\cdots ,m\right)$. The standardized multiple linear regression Equation (1) is

(1) $$y_{i}^{\prime }=\sum_{j=1}^{m}b_{j}^{*}{x^{\prime }}_{j}^{}+\varepsilon_{i}$$

where ${y^{\prime }}_{i}∼N(0,1),\varepsilon_{i}∼N(0,1)$ and $\varepsilon_{i}$ is the random error and different $\varepsilon_{i}s$ are independent of each other. Under the least squares estimation method, the canonical equations to solve the path coefficients can be easily obtained as follows (Equation (2)):

(2) $$[\begin{array}{cccc} 1 & r_{12} & \cdots & r_{1m}\\ r_{21} & 1 & \cdots & r_{2m}\\ \vdots & \vdots & & \vdots \\ r_{m1} & r_{m2} & \cdots & 1 \end{array}][\begin{array}{c} b_{1}^{*}\\ b_{2}^{*}\\ \vdots \\ b_{m}^{*} \end{array}]=[\begin{array}{c} r_{1y}\\ r_{2y}\\ \vdots \\ r_{my} \end{array}]\mathrm{or}{\hat{R}}_{xx}b_{j}^{*}={\hat{R}}_{xy}$$

where ${\hat{R}}_{xx}$ is the maximum likelihood estimation of the correlation matrix $R_{xx}$, and ${\hat{R}}_{xy}$ is the correlation matrix of x and $y_{i}$, which is called the total correlation coefficient (the total effect), reflecting the importance of each PA monomer unit to activity. The closer the absolute value of $r_{jy}$ is to 1, the more important the monomer unit $x_{j}$ is to the activity. For example, if the total effect of the PA monomer unit $x_{j}$ is the largest, then this monomer unit is regarded as the most important in all monomer units to activity. In fact, the canonical equations complete the division of total effect by Equation (2). The solved path coefficient $b^{*}={\left(b_{1}{}^{*},b_{2}{}^{*}\cdots ,b_{m}{}^{*}\right)}^{T}$ indicates the direct effect of each PAs monomer unit on activity $y_{i}$. In addition, the indirect effect of monomer unit $x_{j}$ through the correlation monomer unit $x_{t}$ on activity $y_{i}$ can be demonstrated using $r_{jt}b_{t}^{*}(j=1,2,\cdots ,m;t=1,2,\cdots ,m;t\neq j)$. The subdivided results are displayed in Table 4. The detailed subdivided results can fully display the direct and indirect effect of each monomer unit on activity. For a specified monomer unit, if its direct effect is far greater than the total indirect effect from the relevant monomer unit, it indicates that the monomer unit itself directly affects the activity to a large extent. Otherwise, the monomer unit indirectly affects the activity mainly through related monomer units. This subdivision also can be demonstrated visually as shown in Figure 4. It should be noted that the path analysis is conducted on the basis of significant tests of the multiple linear regression equation. In short, these three parameters $\left(b_{j}^{*},r_{jt}b_{t}^{*},r_{jy}\right)$ can not only quantify the complex regulatory mechanism of different monomer units on activity but also rank the monomer unit’s importance affecting activity. The comparison between the sum of indirect effect ($\sum_{\begin{array}{l} t=1\\ t\neq j \end{array}}^{m}r_{jt}b_{t}^{*}$) and direct effect ($b_{j}^{*}$) can also be used to determine the influence mode of monomer units on activity.

### 4.2. Structural Analysis and Activity Determination 

In order to test the utility of the PA structure-activity relationship path analysis model, the data of monomer units and antioxidant and anti-diabetes activities of *Rhodiola crenulata* PAs were selected. These are listed in Table S1 (See Supplement Materials). Six polymerization degrees (RcPs-f1~RcPs-f6) are considered in this test, and six repetitions are set under each polymerization degree.

High-performance liquid chromatography-electrospray ionization tandem mass spectrometry (HPLC-ESI/MS^2^) and matrix-assisted laser desorption/ionization time-of-flight mass spectrometry (MALDI-TOF/MS) were used to characterize the structure profile (terminal units, extension units, mDP) of PAs in *Rhodiola crenulata*. The antioxidant property of *Rhodiola crenulata* PAs was determined by Ferric reducing antioxidant activity (FRAP) and cupric ion reducing antioxidant capacity (CUPRAC) according to the procedure described by Li et al. [37]. The anti-diabetic capabilities of RCPS were determined by the inhibition of *Saccharomyces cerevisiae* α-glucosidase, porcine pancreatic α-amylase, and human salivary α-amylase, as described previously [37]. 

It Is obvious from the original data that the magnitude difference between monomer unit data and activity data is large, especially for the antioxidant bioactivity data, so data preprocessing is done first before using the path analysis model (See Supplement Material File S1). The processed data used for model analysis are listed in Table S2 (See Supplement Material).

In the path analysis model of the structure-activity relationship of PAs, the monomer units of *Rhodiola crenulata* PAs were selected as independent variables, including terminal units Epicatechin (ECt) $\left(x_{1}\right)$, Catechin gallate (CGt) $\left(x_{2}\right)$, Epicatechin gallate (ECGt) $\left(x_{3}\right)$ and extension units Gallocatechin (GCe) $\left(x_{4}\right)$, Epigallocatechin (EGCe) $\left(x_{5}\right)$, Gallocatechin gallate (GCGe) $\left(x_{6}\right)$, Epigallocatechin gallate (EGCGe) $\left(x_{7}\right)$, Catechin (Ce) $\left(x_{8}\right)$, Epicatechin (ECe) $\left(x_{9}\right)$, and Epicatechin gallate (ECGe) $\left(x_{10}\right)$. The antioxidant and anti-diabetes activities were selected as dependent variables $\left(y_{i},i=1,2\right)$. It should be noted that the degree of polymerization is not considered in the model analysis in order to meet the data size requirements of the model analysis. 

### 4.3. Materials

*Rhodiola crenulata* rhizomes were collected from Lhasa (Tibet, China). PAs extracted from *Rhodiola crenulata* rhizomes were named as RcPs. *Saccharomyces cerevisiae* α-glucosidase (type I, from *Saccharomyces cerevisiae*), α-amylase (from porcine pancreas), α-amylase (from human saliva), 6-hydroxy-2,5,7,8-tetramethylchromo-2-carboxylic acid (Trolox), 2,4,6-tripyridinyl-1,3,5-triazine (TPTZ), dimethyl sulfoxide (DMSO), 4-nitrophenyl-α-D-glucopyranoside (P-NPG), benzyl mercaptan, trifluoroacetic acid (TFA), catechin, epicatechin, epicatechin gallate, epigallocatechin gallate, and gallic acid were purchased from Sigma-Aldrich Chemical Co. (St. Louis, MO, USA). Sephadex LH-20 was purchased from GE Healthcare Bio-Sciences AB (Uppsala, Sweden). Methanol of chromatographic purity was purchased from TEDIA (Fairfield, OH, USA). 2-choro-4-nitrophenyl-&-galactosyl-maltoside (Gal-g2-α-cnp) was purchased from Toyobo Co., Ltd., (Osaka, Japan). Neocuproine was purchased from Shanghai Aladdin Reagents Co., Ltd. (Shanghai, China). Vitamin C was purchased from Shanghai Yuanye Biotechnology Co., Ltd. (Shanghai, China). Sodium acetate, monobasic potassium phosphate, potassium diammonium phosphate, copper sulfate, methanol, ethanol, acetone, petroleum ether (60~90 °C boiling range), and other pure analytical reagents were purchased from Xilong Science Co., Ltd., (Guangdong, China). 

## 5. Conclusions

Overall, this study indicated that the path analysis model of the PA structure-activity relationship can deeply uncover the influence mechanisms of interaction between different PA monomer units on activity through the subdivision of the total effect. The path analysis model is a promising data mining tool for PA structure-activity relationship research. In fact, the activities of PAs depend on not only the structural differences between the monomer units themselves but also the interactions (i.e., correlations) existing between the different monomer units.

## Figures and Tables

**Table 1:** The detailed subdivided results of the total effect between *Rhodiola crenulata* proanthocyanidin monomer units and antioxidant activity.

Monomeric Units	ECt	CGt	ECGt	GCe	EGCe	GCGe	EGCGe	Ce	ECe	ECGe
The direct effect $\left(b_{j}^{*}\right)$ and indirect effect $\left(r_{jt}b_{t}^{*}\right)$ (j,t=1,2,…,m;j≠t)	**0.3521**	−0.3137	−0.3253	−0.3121	0.1765	0.273	−0.0702	0.3245	0.3251	0.31
	−0.4532	**0.5087**	0.4964	0.3476	−0.4228	−0.4744	0.233	−0.4594	−0.4497	−0.4982
	0.0254	−0.0268	**−0.0274**	−0.0221	0.0203	0.0239	−0.0139	0.0237	0.0233	0.0273
	−0.1795	0.1384	0.1629	**0.2026**	−0.0483	−0.0978	0.0522	−0.1376	−0.1391	−0.1524
	−0.0821	0.136	0.1211	0.039	**−0.1637**	−0.1392	0.0919	−0.0995	−0.0998	−0.13
	0.1237	−0.1488	−0.1392	−0.077	0.1358	**0.1596**	−0.0576	0.1307	0.1386	0.1405
	0.0006	−0.0014	−0.0016	−0.0008	0.0017	0.0011	**−0.0031**	0.0006	0.0001	0.0018
	0.3142	−0.3079	−0.2941	−0.2316	0.2072	0.2792	−0.0633	**0.3409**	0.3079	0.2857
	−0.1416	0.1356	0.1304	0.1053	−0.0936	−0.1332	0.0039	−0.1386	**−0.1534**	−0.1247
	0.9787	−1.0886	−1.1053	−0.8361	0.8832	0.9789	−0.6388	0.9315	0.9036	**1.1117**
Sum of indirect effects	0.5861	−1.4772	−0.9548	−0.9876	0.8601	0.7115	−0.4629	0.5759	1.01	−0.1401
Total effect $\left(r_{jy}\right)$	0.9382	−0.9685	−0.9822	−0.785	0.6964	0.8711	−0.466	0.9168	0.8566	0.9716

**Table 2:** The detailed subdivided results of the total effect between *Rhodiola crenulata* proanthocyanidin monomer units and anti-diabetes activity.

Monomeric Units	ECt	CGt	ECGt	GCe	EGCe	GCGe	EGCGe	Ce	ECe	ECGe
The direct effect $\left(b_{j}^{*}\right)$ and indirect effect $\left(r_{jt}b_{t}^{*}\right)$ (j,t=1,2,…,m;j≠t)	**0.3837**	−0.3418	−0.3545	−0.34	0.1923	0.2975	−0.0765	0.3536	0.3542	0.3378
	0.5586	**−0.627**	−0.6118	−0.4284	0.5211	0.5847	−0.2872	0.5662	0.5542	0.614
	1.05	−1.109	**−1.1364**	−0.914	0.8405	0.9913	−0.5774	0.9805	0.9661	1.1299
	0.0257	−0.0198	−0.0233	**−0.029**	0.0069	0.014	−0.0075	0.0197	0.0199	0.0218
	−0.0759	0.1258	0.112	0.0361	**−0.1514**	−0.1288	0.085	−0.092	−0.0923	−0.1203
	0.1012	−0.1217	−0.1139	−0.063	0.111	**0.1305**	−0.0471	0.1069	0.1134	0.1149
	−0.0014	0.0032	0.0035	0.0018	−0.0039	−0.0025	**0.007**	−0.0013	−0.0002	−0.004
	0.5587	−0.5475	−0.5231	−0.4118	0.3685	0.4966	−0.1125	**0.6063**	0.5477	0.508
	−0.5354	0.5126	0.493	0.3982	−0.3536	−0.5036	0.0146	−0.5238	**−0.5799**	−0.4713
	−1.1012	1.2249	1.2436	0.9407	−0.9937	−1.1014	0.7188	−1.0481	−1.0167	**−1.2508**
Sum of indirect effects	0.5803	−0.2734	0.2255	−0.7804	0.6891	0.6477	−0.2898	0.3617	1.4463	2.1308
Total effect $\left(r_{jy}\right)$	0.964	−0.9004	−0.9109	−0.8094	0.5377	0.7782	−0.2828	0.968	0.8664	0.88

**Table 3:**
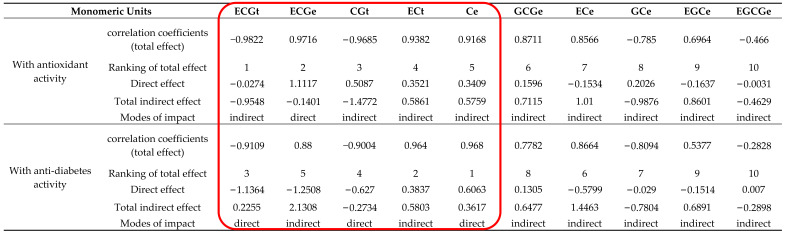
Comparison of the effects of *Rhodiola crenulata* proanthocyanidin monomer units on antioxidant and anti-diabetes activities.

Note: The major monomer units are indicated by the red box.

**Table 4:** The detailed subdivided results of the total effect between monomer units and activity.

Monomeric Units	$x_{1}$	…	$x_{j}$	…	$x_{t}$	…	$x_{m}$
The direct effect $\left(b_{j}^{*}\right)$ and the indirect effect $\left(r_{jt}b_{t}^{*}\right)$ (j,t=1,2,…,m;j≠t)	$\underset{x_{1}↔x_{1}\to y_{i}}{b_{1}^{*}}$	⋯	$\underset{x_{j}↔x_{1}\to y_{i}}{r_{j1}b_{1}^{*}}$	⋯	$\underset{x_{t}↔x_{1}\to y_{i}}{r_{t1}b_{1}^{*}}$	⋯	$\underset{x_{m}↔x_{1}\to y_{i}}{r_{m1}b_{1}^{*}}$
	⋮	⋱	⋮	⋮	⋮	⋮	⋮
	$\underset{x_{1}↔x_{j}\to y_{i}}{r_{1j}b_{j}^{*}}$	⋯	$\underset{x_{j}↔x_{j}\to y_{i}}{b_{j}^{*}}$	⋯	$\underset{x_{t}↔x_{j}\to y_{i}}{r_{tj}b_{j}^{*}}$	⋯	$\underset{x_{m}↔x_{j}\to y_{i}}{r_{mj}b_{j}^{*}}$
	⋮	⋮	⋮	⋱	⋮	⋮	⋮
	$\underset{x_{1}↔x_{t}\to y_{i}}{r_{1t}b_{t}^{*}}$	⋯	$\underset{x_{j}↔x_{t}\to y_{i}}{r_{jt}b_{t}^{*}}$	⋯	$\underset{x_{t}↔x_{t}\to y_{i}}{b_{t}^{*}}$	⋯	$\underset{x_{m}↔x_{t}\to y_{i}}{r_{mt}b_{t}^{*}}$
	⋮	⋮	⋮	⋮	⋮	⋱	⋮
	$\underset{x_{1}↔x_{m}\to y_{i}}{r_{1m}b_{m}^{*}}$	⋯	$\underset{x_{j}↔x_{m}\to y_{i}}{r_{jm}b_{m}^{*}}$	⋯	$\underset{x_{t}↔x_{m}\to y_{i}}{r_{tm}b_{m}^{*}}$	⋯	$\underset{x_{m}↔x_{m}\to y_{i}}{b_{m}^{*}}$
Sum of indirect effects	$\sum_{i=2}^{m}r_{1i}b_{i}^{*}$	⋯	$\sum_{\begin{array}{l} i=1\\ j\neq i \end{array}}^{m}r_{ji}b_{i}^{*}$	⋯	$\sum_{\begin{array}{l} i=1\\ t\neq i \end{array}}^{m}r_{ti}b_{i}^{*}$	⋯	$\sum_{\begin{array}{l} i=1\\ m\neq i \end{array}}^{m}r_{mi}b_{i}^{*}$
Total effect $\left(r_{jy}\right)$	$\underset{x_{1}\to y_{i}}{r_{1y}}$	⋯	$\underset{x_{j}\to y_{i}}{r_{jy}}$	⋯	$\underset{x_{t}\to y_{i}}{r_{ty}}$	⋯	$\underset{x_{m}\to y_{i}}{r_{my}}$

Note: $r_{jt}(j,t=1,2,\ldots ,m;j\neq t)$ indicates the correlation coefficient of $x_{j}$ and $x_{t}$, the data satisfy $r_{jt}=r_{tj}$ and $r_{jy}=b_{j}^{*}+\sum_{\begin{array}{l} t=1\\ t\neq j \end{array}}^{m}r_{jt}b_{t}^{*}$ according to the path analysis model. In order to distinguish between the direct and indirect effects clearly, the direct effect has been indicated in bold italics.

## Data Availability

The data presented in this study are available in supplementary material.

## Supplementary Materials

The following supporting information can be downloaded at: https://www.mdpi.com/article/10.3390/ijms24076379/s1. Table S1: The raw data of monomer units and antioxidant and anti-diabetes activities of *Rhodiola crenulata* PAs; Table S2: The processed data of monomer units and antioxidant and anti-diabetes activities of *Rhodiola crenulata* PAs for model analysis. File S1: The preprocessing method of raw data of monomer units and antioxidant and anti-diabetes activities of *Rhodiola crenulata* PAs.

## Abbreviation

C	Catechin
EC	Epicatechin
EGC	Epigallocatechin
ECG	Epicatechin gallate
EGCG	Epigallocatechin Gallate
