# Supplementary material for: Proanthocyanidin Structure-Activity Relationship Analysis by Path Analysis Model
Source: Int J Mol Sci. 2023 Mar 28;24(7):6379. doi: 10.3390/ijms24076379 (PMC10094556; doi:10.3390/ijms24076379)
Supplement: Supplementary file 1 [file ijms-24-06379-s001.zip › Supplementary Material File S1.pdf]

File S1: The preprocessing method of raw data of monomer units and antioxidant and anti-diabetes activities of Rhodiola crenulata PAs

The antioxidant capability of each sample was determined by FRAP assay and CUPRAC assay. The anti-diabetic capability was determined based on the inhibitory rates of three different enzymes, *S.cerevisiae* α-glucosidase, porcine pancreatic α-amylase, and human salivary α-amylase.

To better illustrate the variance, and comprehensively considering the methods of activity determination, the values from different determination methods were integrated through weighted summation, where the weights were given according to the standard deviation of each evaluation method. The integration results were named integrated antioxidant capability and integrated anti-diabetic capability, which were utilized for further analysis.

We will demonstrate this method using the construction of the integrated antioxidant capability index y_1 as an example.

Firstly, we standardized the two columns of determination results $Data_i = (D_{i1}, D_{i2}, \cdots, D_{im}) \quad (i = 1, 2)$ as follows (equation 1)

$$data_i = (d_{i1}, d_{i2}, \cdots, d_{im})$$
$$d_{ij} = \frac{D_{ij} - D_{i\min}}{D_{i\max} - D_{i\min}}, \qquad (1)$$

where $data_i$ is the standardized data of $Data_i$, D_{imax} indicates the maximum in $Data_i$, while the minimum is indicated as D_{imin}. Then the weight w_i of $data_i$ is defined as equation (2)

$$w_i = \frac{\sigma(data_i)}{\sum_{k=1}^{2}\sigma(data_k)} \tag{2}$$

where $\sigma(data_i)$ is the standard deviation of $data_i$.

Finally, the calculation of $y_1 = (y_{11}, y_{12}, \cdots y_{1m})$ is equation (3)

$$y_{1j} = \sum_{i=1}^{2} w_i \cdot d_{ij} \quad (j = 1, 2, \cdots, m). \tag{3}$$

Similarly, the construction of integrated anti-diabetic capability y_2 is defined in the same way. It should be noted that the values of the three anti-diabetic activity measuring methods are inversely proportional to the activity. Thus, to better illustrate the strength of the activity through values, reciprocal transformation is applied before construction.
